# More than alternative estrogen receptors: the emerging role of GPER-1 and ERα36 in breast cancer

**DOI:** 10.37349/etat.2026.1002377

**Published:** 2026-07-06

**Authors:** Luis Molina Calistro, Rodrigo Flavio Torres, Johana Spies, Sonia Sánchez Meneses, María Soto, Joaquín Carrasco, Javiera Gálvez, Dayanara Muñoz, Javiera Soto, Yennyfer Arancibia

**Affiliations:** IRCCS Istituto Romagnolo per lo Studio dei Tumori (IRST) “Dino Amadori”, Italy; ^1^Facultad de Ciencias, Universidad San Sebastián, Lago Panguipulli 1390, Puerto Montt 5501842, Chile; ^2^Posgrado en Ciencias Biológicas UATx, Centro Tlaxcala Biología de la Conducta, Doctorado en Ciencias Biológicas, Universidad Autónoma de Tlaxcala, Tlaxcala, Tlax 90800, México; ^3^Facultad de Ciencias para el Cuidado de la Salud, Universidad San Sebastián, Puerto Montt 5501842, Chile; ^4^Millennium Nucleus of Neuroepigenetics and Plasticity (EpiNeuro), Santiago 8320000, Chile; ^5^High Altitude Medicine Research Center (CEIMA), Arturo Prat University, Iquique 1110939, Chile

**Keywords:** GPER-1, ERα36, non-genomic estrogen signaling, endocrine resistance, breast cancer subtypes, breast cancer heterogeneity, biomarkers, BPA

## Abstract

Breast cancer classification and therapeutic decision-making have traditionally relied on the evaluation of estrogen receptor alpha (ERα), PR, and HER2, yet this framework does not fully explain tumor heterogeneity, endocrine resistance, or estrogen responsiveness in ERα-negative contexts. Emerging evidence implicates non-genomic estrogen signaling mediated by membrane-associated receptors such as G protein-coupled estrogen receptor 1 (GPER-1) and ERα36. Acting as interconnected signaling nodes, these receptors activate MAPK/ERK and PI3K/AKT pathways and engage in crosstalk with receptors such as EGFR, promoting proliferation, cellular plasticity, and adaptive responses. Here, we propose an integrative framework based on three axes: endocrine resistance in ERα-positive tumors, estrogen responsiveness in ERα-negative subtypes, and environmental modulation of signaling. Within this model, GPER-1 and ERα36 form a coordinated network that extends beyond genomic mechanisms and converges on shared downstream effectors. These pathways also intersect with post-transcriptional regulation, tumor-microenvironment interactions, and extracellular vesicle-mediated communication, contributing to tumor progression and metastasis. Environmental ligands, such as bisphenol A, may further modulate signaling intensity, reinforcing plasticity and resistance phenotypes. Collectively, GPER-1 and ERα36 emerge as candidate biomarkers with diagnostic and therapeutic relevance. Their integration into multi-omics and functional classification strategies may refine breast cancer stratification and support more precise therapeutic approaches.

## Introduction

Breast cancer is currently the most common cancer among women worldwide and remains the leading cause of cancer-related death in women [1]. The increasing incidence of this disease, together with the emergence of primary and acquired resistance to endocrine therapies such as tamoxifen (TMX) and other selective estrogen receptor modulators (SERMs), highlights important limitations in current classification systems based solely on canonical estrogen receptor alpha (ERα) signaling [1]. Mechanistic insights from experimental and clinical studies indicate that resistance to TMX involves complex signaling networks beyond classical ERα activity [2]. Moreover, it is estimated that between 80% and 90% of breast cancer cases are sporadic, supporting a multifactorial etiology rather than a hereditary origin [3, 4].

Despite major advances in biomarker-driven oncology, breast cancer classification remains limited by its reliance on receptor expression, which does not fully capture functional signaling activity or tumor adaptability [5].

From a translational perspective, these limitations highlight a significant gap in biomarker-based oncology, where clinical validation and the implementation of therapeutic targets often lag behind mechanistic advances [6]. In this context, the systematic integration of emerging signaling pathways into diagnostic and therapeutic decision-making is becoming increasingly important, recognizing molecular heterogeneity as a central axis for improving clinical outcomes [7].

Accumulating evidence indicates that estrogen signaling extends beyond the classical nuclear receptor ERα, with membrane-initiated pathways contributing significantly to tumor behavior and therapeutic response [8–10]. A central unresolved issue in breast cancer is the persistence of estrogen-responsive signaling in ERα-negative tumors, as well as the emergence of endocrine resistance in ERα-positive disease [8, 11]. Collectively, these findings underscore a disconnect between clinically defined receptor status and functional estrogen-dependent signaling activity.

This scenario has intensified interest in environmental factors as modulators of breast cancer risk and progression. In particular, chronic exposure to endocrine disruptors such as bisphenol A (BPA) has emerged as a relevant factor due to its ability to interfere with hormonal signaling and reprogram cellular responses associated with carcinogenesis [12, 13]. Importantly, experimental evidence demonstrates that loss of BRCA1 function increases cellular sensitivity to BPA, suggesting a gene-environment interaction in breast cancer susceptibility [14].

Furthermore, BPA and its analogues have been shown to alter mammary epithelial morphogenesis, reinforcing their potential role in early tumorigenic processes [15]. The widespread presence of BPA and its structural analogues, along with their estrogenic activity at low doses, strengthens their potential impact on breast tumor biology [16].

Rather than acting as independent etiological agents, environmental estrogens such as BPA are increasingly understood as modulators of estrogen signaling networks, particularly those mediated by membrane-associated receptors, thereby influencing signaling amplitude and cellular responsiveness [17]. Therefore, environmental exposures should be considered within the framework of the complexity of estrogen signaling, rather than as an isolated carcinogenic factor.

Clinically, approximately 70% of breast cancers are classified as hormone-sensitive based on the expression of ERα and/or PR [18]. In contrast, the absence of these receptors, along with the lack of HER2, defines triple-negative breast cancer (TNBC), one of the most aggressive subtypes with limited therapeutic options [19]. However, this classification does not fully explain tumor heterogeneity, the variability in therapeutic response, or the paradoxical response to estrogens observed in a subset of ERα-negative tumors [19]. This limitation suggests that other pathways may contribute to the estrogen response beyond ERα detection.

In this context, recent reviews have highlighted the relevance of G protein-coupled estrogen receptor 1 (GPER-1) in ERα-negative breast cancer, supporting its role as an alternative mediator of estrogen signaling in these tumors [20]. Complementarily, the ERα36 isoform has emerged as a critical determinant of non-genomic estrogen signaling and endocrine resistance [21].

Both GPER-1 and ERα36 have been consistently associated with rapid signaling activation and resistance-related phenotypes in experimental models. These receptors can be activated not only by endogenous estrogens but also by xenoestrogens and endocrine disruptors, including bisphenols such as BPA [22–24]. Furthermore, ERα36 has been experimentally linked to TMX resistance and enhanced metastatic behavior in breast cancer cell models, supporting its functional relevance in disease progression [25].

Importantly, most of the mechanistic evidence supporting these roles derives from experimental models, particularly breast cancer cell lines and in vivo systems, and therefore should be interpreted within this context when extrapolating to clinical scenarios. Notably, both GPER-1 and ERα36 converge on key signaling pathways involved in proliferation, survival, migration, invasion, and endocrine resistance [26]. These pathways include major proliferative and survival cascades such as MAPK/ERK and PI3K/AKT, which are recurrently activated downstream of non-canonical estrogen receptors [27, 28].

Furthermore, although these pathways are initiated at the membrane level, they converge on transcriptional regulators, reinforcing the concept of a functional continuum between rapid signaling and gene expression control. Importantly, although these pathways are frequently described independently, they largely converge on shared intracellular signaling nodes, suggesting that receptor-specific activation may lead to overlapping phenotypic outcomes [20].

Additionally, variations in estrogen receptor expression patterns in tumor tissues have been associated with clinical parameters such as hormone levels and prognostic indices, underscoring the importance of receptor heterogeneity in disease outcome [29]. The heterogeneity of receptors should not be understood solely in terms of the presence or absence of ERα, but rather as a spectrum that includes alternative isoforms and membrane-associated receptors [30]. In this context, the variability observed in ERα-positive tumors reinforces the need to incorporate membrane-bound estrogen receptors into current biological models, not as parallel entities, but as integral components of the molecular heterogeneity that shapes estrogen signaling [31].

Importantly, receptor isoform expression and signaling plasticity are also influenced by post-transcriptional regulatory mechanisms that expand transcript diversity and functional adaptability [32]. Among these, RNA editing enzymes such as ADAR1, whose expression varies according to ER status and is associated with prognostic outcomes in breast cancer, can reshape RNA sequences, affecting mRNA stability, splicing, and coding potential [33]. Through these effects, ADAR1 may modulate the repertoire of receptor isoforms and downstream signaling pathways, facilitating adaptive responses to endocrine therapies and contributing to the development of resistance [34, 35].

Despite the growing body of literature addressing individual aspects of non-genomic estrogen signaling, a comprehensive framework integrating membrane-associated estrogen receptors with environmental modulation and tumor adaptive plasticity remains lacking.

To address these limitations, this review adopts an integrative framework organized around three interrelated axes: (i) endocrine resistance in ERα-positive tumors, (ii) estrogen responsiveness in ERα-negative contexts, and (iii) environmental modulation of non-genomic estrogen signaling.

This approach aims to bridge mechanistic insights with translational relevance, positioning non-genomic estrogen receptors within the broader evolution of biomarker-driven oncology [36].

## Estrogens and their interaction with classical nuclear receptors

In accordance with the conceptual framework described above, we will now discuss estrogen biology to contextualize the complexity of estrogen signaling in hormone-dependent tumors, without intending to provide an exhaustive review of steroidogenesis.

Estrogens are synthesized from cholesterol through steroidogenesis, primarily in the gonads and, to a lesser extent, in the adrenal cortex. The aromatization of androgens constitutes the final and rate-limiting step in estrogen production [37], determining both systemic and local estrogen availability in tissues such as the breast.

In humans, the main endogenous estrogens—estradiol (E2), estrone (E1), and estriol (E3)—exhibit distinct biological roles and relative abundances depending on physiological context [38]. E2 is the most potent estrogen and predominates during reproductive years, whereas E1 becomes more relevant after menopause due to peripheral aromatization in adipose tissue, linking metabolic status with estrogen exposure [39]. E3, mainly produced during pregnancy, displays weaker estrogenic activity [40].

Endogenous estrogens exert their biological effects through a receptor network that has been central to modern endocrinology. The identification of ERα by Elwood Jensen established the basis for understanding estrogen action [41]. The subsequent discovery of estrogen receptor beta (ERβ) further expanded this framework, revealing a more complex regulatory system [42].

Both ERα and ERβ belong to the nuclear receptor superfamily and function primarily as ligand-activated transcription factors. Upon activation, they bind estrogen response elements (EREs) and regulate gene expression programs involved in proliferation, metabolism, differentiation, and immune modulation [43]. This genomic signaling pathway has provided the foundation for breast cancer classification and the development of endocrine therapies [44].

Despite structural similarities, ERα and ERβ differ in tissue distribution and functional output. ERα is generally associated with proliferative signaling in breast tissue, whereas ERβ is often linked to anti-proliferative and differentiating effects [45]. These differences contribute to the complexity of estrogen signaling in physiological and pathological contexts.

Although classical nuclear signaling has been central to understanding estrogen biology, it does not fully account for the diversity, rapid kinetics, and context-dependent nature of estrogen responses. This limitation is particularly evident in breast cancer, where receptor status does not consistently predict functional signaling activity or therapeutic response, partly due to pathway crosstalk, receptor plasticity, and endocrine resistance mechanisms [46].

Importantly, estrogen signaling is not restricted to ligand-dependent transcriptional regulation. Increasing evidence demonstrates that estrogen responses can also be initiated at the membrane level and transmitted through rapid intracellular cascades [47]. Although distinct in kinetics, these pathways converge on transcriptional regulators, supporting the concept of estrogen signaling as an integrated network in which receptor localization influences signaling dynamics and biological outcomes.

The molecular classification of breast cancer, established through gene expression profiling, defines clinically relevant subtypes such as luminal A, luminal B, HER2-enriched, and TNBC [48]. While this framework remains essential for diagnosis and treatment, it does not fully capture tumor heterogeneity or explain variability in therapeutic response [49, 50].

These limitations underscore a broader challenge in biomarker-driven classification, where receptor expression alone may be insufficient to capture the functional signaling dynamics within tumor cells.

In particular, estrogen-responsive behaviors observed in ERα-negative tumors and persistent signaling in the absence of detectable nuclear receptors highlight limitations of receptor-based classification systems [8]. These observations reveal a gap between receptor expression and functional signaling activity, supported by experimental and translational studies showing discrepancies between molecular classification and intracellular signaling dynamics [51–53].

Consequently, non-genomic estrogen signaling pathways emerge as crucial mechanisms that broaden our understanding of the functional role of estrogens. Membrane-associated receptors, such as GPER-1 and ERα36, remain functionally active in different breast cancer subtypes and can be activated not only by endogenous estrogens but also by SERMs and environmental ligands [24, 54].

Within this framework, GPER-1 and ERα36 are not considered isolated alternative receptors but rather components of a broader signaling architecture that refines the current classification of subtypes and their therapeutic interpretation.

It is important to note that the identification of membrane-associated estrogen receptors aligns with the overall evolution of biomarker-based oncology, where mechanistic discoveries provide progressively more information about clinically relevant targets, thus reducing the gap between the complexity of molecular signaling and therapeutic decision-making [7].

Overall, while the genomic signaling of estrogen receptors forms the basis of current classification systems, it does not fully encompass the functional diversity of estrogen signaling in breast cancer.

## Rapid estrogen responses: integration of genomic and non-genomic pathways

### The rise of GPER-1, an unconventional estrogen receptor

Within the context of tumor heterogeneity and endocrine resistance, non-genomic estrogen signaling pathways have gained increasing attention as mechanisms that extend beyond classical receptor-mediated transcription. Among these, GPER-1 has emerged as a key mediator of rapid estrogen signaling, linking extracellular stimuli with intracellular adaptive responses [55–57]. This paradigm shift reflects the growing recognition that the classical ERα-centered framework is insufficient to fully explain the diversity and dynamics of estrogen-driven effects in cancer.

Consistent with this perspective, increasing attention has been directed toward rapid estrogen responses that occur independently of transcriptional regulation. Early observations in the late 1990s reported cellular responses to E2 within seconds to minutes, providing initial evidence for signaling pathways operating beyond nuclear receptor activity [58]. Building on these findings, the proposal of an estrogen-sensitive G protein-coupled receptor (GPCR) in 2000 introduced a mechanistic basis for such rapid effects, supporting the existence of a non-genomic signaling route [20]. Shortly thereafter, Filardo and colleagues [59, 60] identified and characterized this receptor, initially termed GPR30 and later renamed GPER-1, establishing its responsiveness to E2 and its role in rapid signaling [61].

GPER-1 is predominantly localized at the plasma membrane, consistent with its classification as a GPCR [62], although it has also been detected in intracellular compartments such as the endoplasmic reticulum and Golgi apparatus [63]. This distribution is not merely descriptive; rather, it is functionally determinant, as receptor localization influences signaling kinetics, pathway engagement, and downstream biological responses. Reports of nuclear localization remain limited and somewhat controversial [57, 64], and in the context of non-genomic signaling should be interpreted cautiously, likely reflecting indirect modulation of transcription rather than canonical receptor activity.

Consistent with this view, the spatial organization of GPER-1 contributes to the regulation of proliferation, migration, and transcriptional responses by shaping pathway selection and signal propagation dynamics [26, 57, 64, 65].

From a broader perspective, GPER-1 shows how membrane-initiated estrogen signaling allows rapid signal propagation, while remaining coupled to transcriptional regulatory networks. This interdependence between subcellular localization and signaling is especially relevant in cancer, where changes in receptor distribution under therapeutic pressure can reconfigure signaling pathways and favor the appearance of more resistant phenotypes [22, 66, 67].

Beyond endogenous ligands, GPER-1 is activated by xenoestrogens such as BPA, a ubiquitous environmental compound detectable in multiple human biological fluids [23, 68, 69]. BPA can interact with membrane-associated estrogen receptors, including GPER-1 and ERα36, even at nanomolar concentrations [70–72]. This ligand promiscuity extends the functional scope of GPER-1 beyond physiological signaling, incorporating environmental modulation into its activity profile [73, 74].

Importantly, although experimental models consistently demonstrate GPER-1 activation by BPA, the magnitude and clinical relevance of these effects remain context-dependent, particularly with respect to exposure timing and tissue susceptibility [75]. In this context, BPA exposure links environmental signals to non-genomic estrogen pathways, reinforcing the need to consider endocrine disruption within broader signaling networks than those commanded exclusively by classical estrogen receptors [76].

At the molecular level, GPER-1 activation engages heterotrimeric G proteins, leading to cAMP production via adenylate cyclase and subsequent activation of protein kinase A [65]. In parallel, Gβγ subunits promote intracellular Ca^2+^ mobilization [77, 78]. Additional signaling involves Src kinase activation, metalloprotease-mediated HB-EGF release, and transactivation of EGFR, ultimately converging on MAPK/ERK and PI3K/AKT pathways [79].

These cascades are central to cancer biology, as they integrate extracellular cues with proliferation, survival, and migration programs [80]. Furthermore, these pathways converge on shared intracellular nodes that regulate both rapid signaling and transcriptional programs [81, 82]. This convergence reinforces the concept that non-genomic signaling is functionally integrated with transcriptional regulation.

Within this integrated network, additional regulatory layers include L-type calcium channels (e.g., Cav1.3) and integrin-mediated adhesion complexes, which further amplify Ca^2+^ signaling and EGFR transactivation [83–85]. Collectively, these interconnected pathways support cellular plasticity, metabolic adaptation, and survival [68].

Pharmacological and genetic approaches, including selective agonists (G1), antagonists (G15, G36), and gene silencing, have validated the functional relevance of GPER-1 [86, 87]. Experimental models further demonstrate its involvement in vascular regulation, inflammation, and tumor-associated processes such as proliferation and migration [88–91].

In breast cancer, GPER-1 has been implicated in endocrine resistance. In both E2-responsive and TMX-treated cells, its activation promotes MAPK/ERK and PI3K/AKT signaling [91–93]. Prolonged TMX exposure increases GPER-1 expression and enhances Ca^2+^ mobilization, leading to increased proliferation, while resistant models show enhanced membrane localization and crosstalk with EGFR [92, 93].

The interaction of GPER-1-dependent estrogen signaling pathways allows for adaptive reorganization that is particularly relevant in the context of endocrine resistance, where sustained activation of the pathways compensates for the inhibition of ERα signaling [22, 67, 94].

Clinically, GPER-1 expression has been associated with poor response to TMX, reduced relapse-free survival, and correlations with EGFR, HER2, and lymph node involvement [22, 95, 96]. These observations, supported by experimental, clinical, and computational data, reinforce its relevance as a potential prognostic biomarker [97]. This convergence supports the concept that ERα-negative tumors may retain functional estrogen responsiveness through non-genomic receptors [8].

Beyond tumor cells, GPER-1 also modulates the tumor microenvironment. In cancer-associated fibroblasts, its activation induces HMGB1 secretion, promoting autophagy and TMX resistance [98]. Large-scale analyses (TCGA, METABRIC) further associate high GPER-1 expression with pro-metastatic pathways and adverse outcomes, particularly in ERα-negative and TNBC [97, 99].

In TNBC models, inhibition of GPER-1 reduces proliferation, migration, ERK/AKT activation, tumor growth, and metastasis [22, 91, 100, 101]. It also promotes epithelial-mesenchymal transition (EMT), stemness, and adaptation to hypoxia via HIF-1α, further supporting its role in tumor plasticity [26, 102, 103].

Overall, GPER-1 should be understood not merely as a mediator of rapid estrogen signaling but as an integrative node linking extracellular stimuli, intracellular kinase networks, and transcriptional outputs within dynamic signaling systems.

### ERα36, transcending the classical paradigm of the nuclear receptor

In parallel with GPER-1, the identification of ERα36 has further expanded the conceptual framework of estrogen signaling. ERα36 is an alternative splice variant of the *ESR1* gene encoding a 36 kDa protein that lacks the AF-1 and AF-2 transcriptional activation domains [104, 105]. Although it retains DNA- and ligand-binding regions, it does not function as a classical transcription factor. Instead, it is primarily localized to the plasma membrane and cytoplasm, particularly in caveolae, where it mediates rapid signaling [104].

ERα36 interacts with EGFR and other membrane-associated complexes, activating MAPK/ERK, PI3K/AKT, Src/FAK, and PKC pathways, thereby promoting proliferation, migration, and invasion [105–107].

Consistent with its structural features, ERα36 is best understood as a mediator of membrane-initiated signaling whose biological effects depend on integration within broader signaling networks rather than direct transcriptional regulation [108]. This conceptual shift is essential to reposition ERα36 within estrogen signaling, moving from a truncated receptor view toward a systems-level regulator of signaling dynamics.

Although occasional nuclear localization has been reported, it is associated with aggressive phenotypes and likely reflects indirect modulation of transcription rather than canonical receptor activity [21]. Functional studies using RNA interference demonstrate that ERα36 contributes to proliferation, EMT, stemness, and TMX resistance [107, 109]. However, selective targeting remains technically challenging due to shared exons with canonical ERα, a limitation that may contribute to inter-study variability [24].

In ER-positive models, ERα36 interferes with ERα signaling and redirects estrogen responses toward non-genomic pathways [24, 110]. Notably, TMX can act as an agonist of ERα36, activating EGFR/ERK signaling and promoting proliferation [111]. Inhibition of EGFR/HER2 or ERα36-associated pathways restores TMX sensitivity, including in stem-like cell populations [112]. These findings support a model in which ERα36 redirects signaling during endocrine therapy, contributing to resistance. This redirection mechanism may provide an explanation for the paradoxical effects of TMX observed in certain tumor contexts.

Importantly, ERα36 expression should be interpreted within a broader regulatory landscape that includes post-transcriptional mechanisms such as RNA editing [24]. Enzymes like ADAR1 have been associated with breast cancer heterogeneity and prognosis, supporting the concept that receptor isoform expression and signaling plasticity are shaped by multilayer regulatory processes [113]. In this line, ERα36 upregulation would represent a biologically coherent extension of intrinsic tumor heterogeneity rather than an isolated alteration.

Clinically, ERα36 overexpression is associated with poor disease-free survival and reduced response to TMX [111, 114]. Approximately 44% of ERα-positive tumors overexpress ERα36, correlating with increased metastatic potential and therapeutic resistance [115, 116]. Systems-level analyses further suggest that ERα36 promotes transitions toward more aggressive, endocrine-resistant phenotypes [117].

In TNBC models, ERα36 mediates rapid responses to E2 and environmental estrogens such as BPA, activating MAPK/ERK, PI3K/AKT, Src/FAK, and EGFR pathways [21, 106]. Silencing ERα36 reduces tumor growth in xenograft models, supporting its functional relevance [106]. In HER2-positive tumors, ERα36 also contributes to adaptive resistance through crosstalk with EGFR and HER2 [24].

ERα36 further integrates EMT and stemness programs through MAPK/ERK, PI3K/AKT, and Wnt/β-catenin signaling. In this context, YAP emerges as a convergent effector linking mechanical and mitogenic cues with transcriptional programs associated with plasticity and self-renewal [118–120].

Importantly, this positioning of ERα36 within multiple convergent pathways reinforces its role as a signaling hub rather than a linear pathway component. Within this integrated network, ERα36 contributes to tumor heterogeneity by coordinating proliferative, survival, and stemness-related programs [21, 107, 117].

Together with GPER-1, ERα36 supports an integrated model in which membrane-initiated signaling dynamically interacts with transcriptional regulation. This framework provides a mechanistic basis for estrogen signaling in ERα-negative tumors and opens opportunities for the identification of new therapeutic targets.

## Implications of GPER-1/ERα36 coexpression in the development of breast cancer

### Expanding non-genomic estrogen signaling across cancer types

Building on the integrated model of estrogen signaling outlined above, the functional relevance of GPER-1 and ERα36 extends beyond rapid, membrane-initiated responses to include broader regulatory effects on transcriptional programs and cellular adaptation. Although both receptors are predominantly localized at the plasma membrane, alternative subcellular distributions -such as cytoplasmic and, less consistently, nuclear localization- have also been described. Beyond canonical trafficking through the endoplasmic reticulum and Golgi apparatus, emerging evidence indicates that alterations in receptor localization and intracellular trafficking can influence the functional availability of membrane-associated estrogen receptors, thereby modulating downstream signaling outputs in cancer [44, 121, 122]. This variability highlights the dynamic and context-dependent nature of non-canonical estrogen receptors.

**Figure 1:**
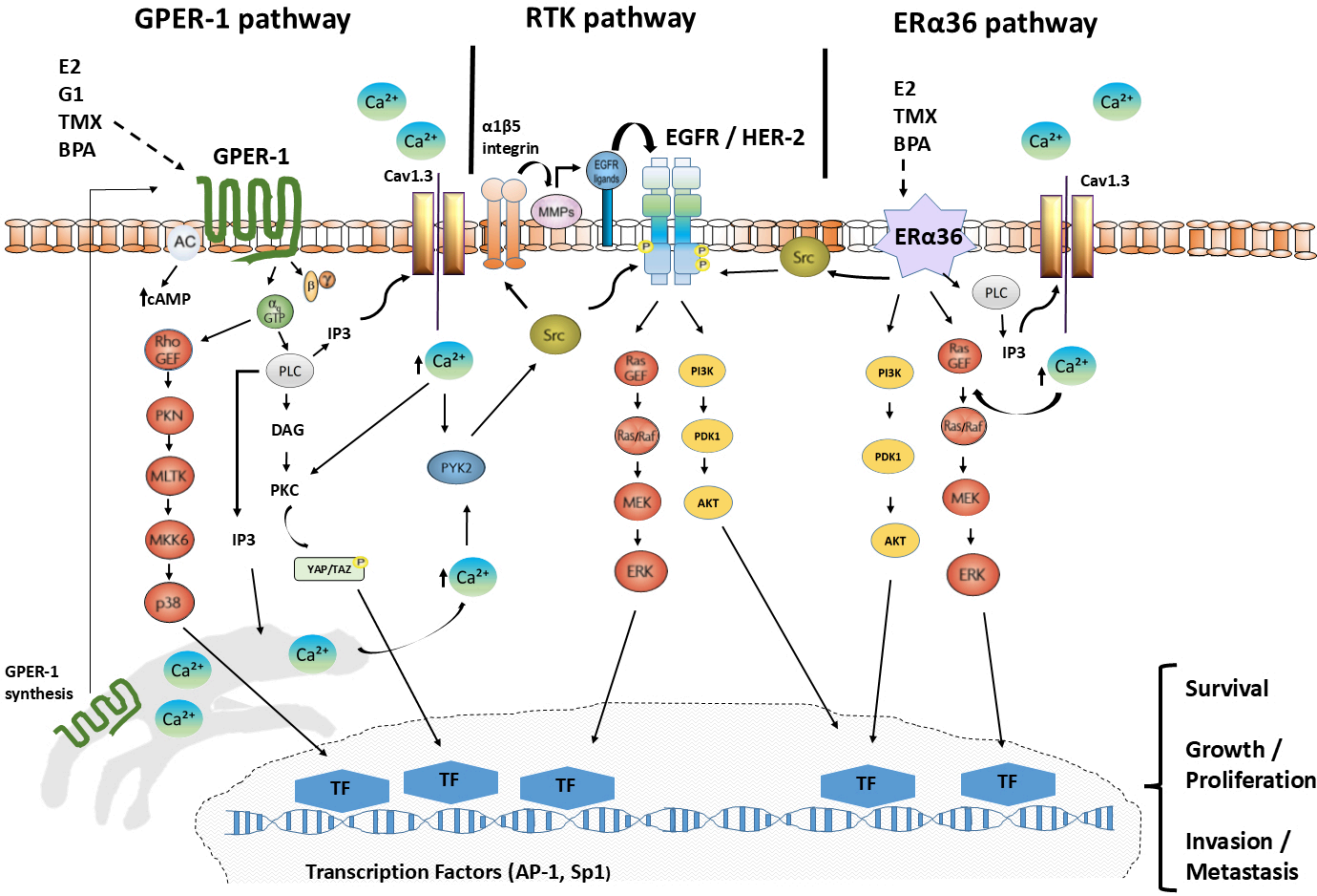
**Node of convergence between GPER-1, RTKs, and ERα36 in breast cancer.** GPER-1 and ERα36 functionally converge with tyrosine kinase receptors (RTKs), especially EGFR/HER2, activating Src and rapid signaling pathways that include intracellular Ca^2+^ mobilization, MAPK/ERK, PI3K/AKT, and YAP/TAZ, which promote tumor plasticity and EMT. These responses are associated with therapeutic resistance in breast cancer.

Importantly, signaling downstream of GPER-1 and ERα36 is not restricted to transient cytoplasmic events. Multiple effectors activated by these receptors converge on transcriptional regulators associated with proliferation, survival, and adaptation [123]. This convergence establishes a mechanistic link between rapid signaling and transcriptional reprogramming in cancer cells, supporting a model in which membrane-initiated estrogen signaling operates as part of a functional continuum rather than as an isolated non-genomic process (Figure 1).

As such, early signaling events can be translated into sustained gene expression changes [124], a feature that is particularly relevant in cancer, where transient stimuli may consolidate into stable phenotypic states, promoting cellular plasticity [125, 126] and therapeutic resistance [127].

The activity of these receptors has been documented across multiple physiological and pathological contexts. In cardiovascular systems, GPER-1 and ERα36 regulate endothelial and vascular smooth muscle function, influencing vascular tone and inflammatory responses [128–130]. In the nervous system, they contribute to neuronal survival, synaptic plasticity, oxidative stress responses, and neuroprotection [131, 132]. In metabolic tissues, they modulate insulin sensitivity and energy homeostasis [133].

Taken together, these observations support the notion that non-genomic estrogen signaling represents a conserved regulatory mechanism across tissues [134, 135], reinforcing its biological relevance in cancer development [134].

In this way, non-genomic estrogen signaling should be interpreted not as a secondary or accessory pathway, but as a fundamental component of estrogen biology with conserved roles in cell proliferation, survival, and adaptation across physiological and pathological conditions (Figure 1).

Consistently, in proliferative benign conditions such as endometriosis and uterine leiomyomas, GPER-1 and ERα36 promote survival and tissue expansion [71, 136]. In hormone-related cancers, including endometrial and ovarian tumors, their expression has been associated with sustained proliferative and migratory responses [111, 133].

Altogether, this cross-tissue evidence supports a model in which GPER-1 and ERα36 act as conserved mediators of estrogen signaling, whose dysregulation in cancer contributes to sustained proliferative signaling, cellular plasticity, and therapeutic resistance.

### Evidence and limitations of GPER-1/ERα36 co-expression

Despite the growing interest in non-genomic estrogen receptors, no clinical studies have directly evaluated the simultaneous coexpression of GPER-1 and ERα36 in breast cancer. Nevertheless, indirect evidence supports the plausibility of their coexistence. GPER-1 is detected in approximately 50–60% of tumors [93] and co-expressed with ERα in around 40% of cases [122, 137]. ERα36 is present in roughly 40% of breast cancers, including luminal B and triple-negative subtypes [24, 138, 139].

Taken together, these prevalence patterns suggest that co-expression may occur in a substantial subset of tumors. However, such estimates should be interpreted with caution, as they are not derived from studies specifically designed to assess both receptors simultaneously.

This gap highlights the need for prospective studies specifically designed to assess co-expression patterns and their clinical implications. In particular, the absence of integrated multi-omics datasets that simultaneously evaluate both receptors limits the ability to accurately characterize their coordinated activity and to understand how this interaction shapes tumor behavior.

### GPER-1/ERα36 crosstalk: functional interaction and amplification of adaptive signaling in cancer

From an evolutionary and functional perspective, crosstalk between estrogen receptors provides signaling versatility [134, 140, 141]. Experimental evidence supports this interaction: in heterologous systems, GPER-1 overexpression has been associated with modulation of ERα36-related signaling, suggesting a functional interplay between membrane-initiated and non-classical estrogen receptor pathways rather than a consistent direct regulation of receptor expression [142]. Interestingly, both GPER-1 and ERα36 activate HER2/EGFR by triggering the activation of MAPK/ERK and PI3K/AKT [24, 143–145]. This circuit can establish a cross-talk that promotes tumor development in different types of breast cancer (Figure 1).

These findings support a regulatory interaction between GPER-1 and ERα36 that may establish a positive feedback loop [24, 127], enhancing non-genomic estrogen signaling and promoting adaptive plasticity [24]. In this context, such a loop could sustain pathway activation under selective pressures, including endocrine-disrupting chemicals (EDCs) and endocrine therapy [146, 147].

Additionally, interactions between GPER-1 and ERα signaling pathways have been shown to modulate inflammatory responses, including inhibition of NF-κB activity and regulation of cytokines such as TNFα and IL-6, suggesting a role in shaping the tumor microenvironment [121]. These observations extend estrogen signaling to tumor-microenvironment communication, although this dimension remains incompletely characterized.

Beyond intracellular signaling, non-genomic estrogen receptors may also play a relevant role in intercellular communication within the tumor microenvironment [148]. Indeed, extracellular vesicles (EVs) have emerged as key mediators of signal propagation, enabling the transfer of proteins, lipids, and nucleic acids between tumor and stromal cells. EV-mediated cargo transfer can thus contribute to EMT, stemness, and metastatic progression [149].

Within this framework, the available evidence supports a plausible mechanistic link between membrane estrogen receptor activation and EV biogenesis [150]. As established, E2 stimulates exosome biogenesis through nSMase2 activation, promoting ceramide production and intraluminal vesicle formation via an ESCRT-independent pathway, thereby increasing EV release with potential implications for tumor progression [151]. GPER-1 stimulation triggers rapid signaling pathways, including PLC/IP3-mediated Ca^2+^ mobilization, and is functionally associated with intracellular trafficking routes involving the endoplasmic reticulum-Golgi axis and endocytic processes, mechanisms closely linked to membrane dynamics and vesicular transport [152, 153]. Moreover, studies on GPCRs indicate that these pathways can regulate EV release and that the receptors themselves may be incorporated as functional cargo, facilitating intercellular signal propagation [154, 155]. Collectively, these findings support a model in which GPER-1 may integrate into EV-associated pathways, contributing to the dissemination of non-genomic estrogen signaling and potentially to tumor adaptive responses [156].

In parallel, evidence suggests that ERα36 is also connected to pathways regulating membrane dynamics and vesicular trafficking. ERα36 mediates rapid non-genomic signaling through activation of the MAPK/ERK and PI3K/AKT cascades [27], which are closely associated with cytoskeletal remodeling, endocytosis, and intracellular transport [109]. These features position ERα36 within signaling networks compatible with vesicle formation and trafficking. Although direct evidence linking ERα36 to EV biogenesis remains limited, its role in rapid signaling, cellular plasticity, and endocrine resistance supports its potential involvement in EV-mediated intercellular communication, facilitating the propagation of non-genomic estrogenic signals and adaptive tumor responses.

However, this highlights analogies with other GPCRs and signaling systems rather than from direct experimental demonstrations linking GPER-1 or ERα36 activation to EV biogenesis or cargo composition [154, 157, 158]. Therefore, specific functional studies are required to directly validate the participation of these receptors in EV-related processes within relevant pathophysiological contexts.

**Figure 2:**
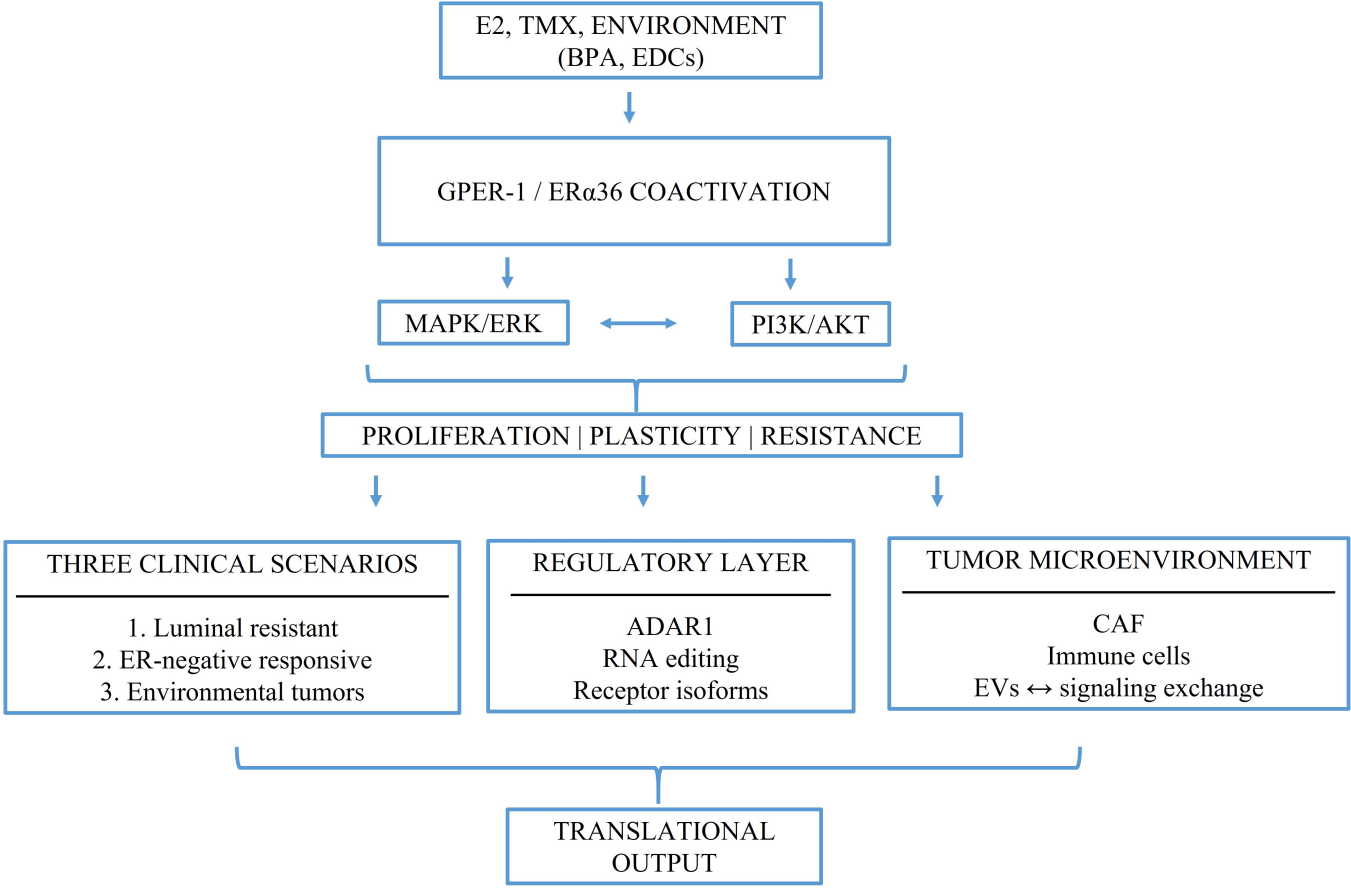
**The integrative framework GPER-1/ERα36 coactivation.** GPER-1 and ERα36 coactivation connects endocrine resistance, ERα-negative estrogen responsiveness, and environmental modulation of signaling. The model also incorporates tumor-microenvironment interactions, extracellular vesicle-mediated communication, and multi-omics-based translational applications.

In perspective, the interaction between GPER-1 and ERα36 likely amplifies non-genomic estrogen signaling and functionally integrates tumor cells with their microenvironment, potentially through feedback mechanisms that promote adaptive plasticity [54, 121, 159]. Furthermore, their association with vesicular trafficking pathways suggests a possible role in EV-mediated communication (Figure 2).

On the other hand, there is a clear need for prospective studies that not only evaluate co-expression patterns but also define their clinical relevance.

These findings support a model in which non-genomic estrogen signaling pathways—particularly those mediated by GPER-1 and ERα36—functionally complement classical receptor systems. To integrate these observations into a clinically applicable framework, we propose a multi-marker panel incorporating ERα, PR, EGFR, GPER-1, and ERα36 (Table 1).

**Table 1:** **GPER-1 and ERα36 in expanded ERα/PR/EGFR biomarker frameworks**.

**Biomarker**	**Role**	**Evidence type**	**Tumor evidence**	**Subtype**	**Functional implication**	**Diagnostic value**	**Main references**
**ERα**	Classical nuclear estrogen receptor	Primary experimental + clinical + genomic	Defines luminal tumors	Luminal A/B	Hormone-dependent transcriptional regulation	Core clinical marker	Clinical standard
**PR**	ERα downstream effector	Clinical + mechanistic	ER axis integrity	Luminal A/B	Functional ER signaling	Predicts endocrine response	Clinical standard
**EGFR**	RTK	Experimental + clinical	Aggressive tumors	TNBC/HER2	MAPK/PI3K signaling	Aggressive marker	[10, 27, 79, 86, 106, 160]
**GPER-1**	GPCR non-genomic	Experimental + pharmacology + translational	~50–60% tumors	Luminal resistant/TNBC	Plasticity, resistance	ER-independent signaling	[22, 26, 28, 56, 66, 70, 85, 86, 90, 92, 93, 95–99, 101–103, 135, 160, 161]
**ERα36**	Splice variant ESR1	Experimental + translational	~30–40% tumors	Luminal B/TNBC	EMT, resistance	Non-classical ER signaling	[10, 21, 25, 27, 104–106, 108–112, 114–117, 139]
**GPER-1 +** **ERα36**	Crosstalk axis	Mechanistic integration	Not unified clinically	Luminal resistant/TNBC	Adaptive plasticity	Candidate axis	[24, 29, 54, 93, 121, 127]
**Integration**	Network signaling	Systems biology	Correlative datasets	All	MAPK/PI3K-transcription	Multi-marker support	[29, 79, 81, 97, 117, 143, 144, 162]
**Limitation**	Translational gap	Methodological	Limited validation	—	Context dependence	Needs clinical validation	[6, 163]

### Environmental modulation and sporadic breast cancer

The relevance of a GPER-1/ERα36 signaling axis becomes particularly evident in the context of sporadic breast cancer and environmental exposure [164]. Within this mechanistic framework, membrane-associated estrogen receptors exhibit responsiveness to non-classical ligands, including EDCs such as BPA. Despite its relatively low affinity for classical ERα, BPA efficiently activates non-genomic estrogen signaling pathways mediated by GPER-1 and ERα36 [70–72], thereby providing a biologically plausible link between environmental exposure and tumor signaling plasticity.

These findings support a role for environmental ligands as modulators of non-genomic estrogen signaling. Importantly, while BPA clearly activates non-genomic pathways in experimental systems [165], the extent to which these mechanisms translate into clinically relevant effects depends on exposure levels, timing, and tissue context [165].

Additional experimental evidence further supports the interaction between environmental estrogens and tumor susceptibility pathways. For instance, loss of BRCA1 has been associated with increased cellular sensitivity to BPA exposure, suggesting that genetic background may modulate the impact of endocrine disruptors on breast cancer risk [14]. Moreover, BPA and structurally related compounds such as bisphenol S have been shown to disrupt mammary epithelial cell morphogenesis, reinforcing their potential role in early tumorigenic processes [15].

Within this framework, environmental estrogens are best understood as modulators of signaling intensity rather than primary oncogenic drivers [166, 167].

This perspective is particularly relevant in sporadic breast cancer [166, 167], where chronic low-dose exposure is estimated to progressively remodel estrogen-sensitive signaling networks over time [168, 169].

Although direct binding of BPA to ERα36 remains less clearly established, functional evidence supports the involvement of ERα36 in BPA-induced signaling [24, 71].

Collectively, current evidence supports a model in which environmental estrogens, particularly BPA, act as context-dependent modulators of non-genomic estrogen signaling rather than primary oncogenic drivers [170]. Through activation of GPER-1/ERα36-associated pathways, environmental estrogens such as BPA can enhance proliferative and adaptive signaling, EGFR-mediated responses [70, 160, 171]. These effects are consistent with rapid, non-genomic estrogen signaling mechanisms that support cellular adaptation (Figure 1). Importantly, their overall impact is shaped by exposure dynamics and tissue-specific context [8, 165], contributing to the progressive reprogramming of estrogen-responsive signaling networks over time [70, 172].

Both GPER-1 and ERα36 converge in mechanisms involving intracellular Ca^2+^ and EGFR transactivation [143, 144]. Notably, ERα36 can form membrane-associated complexes with Src to directly activate ERK2 independently of EGFR, whereas GPER-1 can activate ERK1/2 or p38 MAPK through alternative routes (Figure 1). These observations indicate that both receptors engage overlapping but non-identical signaling pathways, enabling both redundancy and diversification of signaling outputs. Rather than operating in a linear hierarchy, GPER-1 and ERα36 are better understood as components of parallel and cooperative signaling modules.

Overall, the available evidence supports the plausibility of functional cooperation between GPER-1 and ERα36 in breast cancer cells. This cooperative framework helps explain the robustness and adaptability of tumor signaling (Figure 2), although further studies are required to directly validate these interactions in clinically relevant contexts.

### Translational relevance of GPER-1/ERα36 coactivation in breast cancer subtypes

Breast cancer exhibits marked biological and clinical heterogeneity that cannot be fully explained by classical estrogen receptor signaling alone [8]. Increasing evidence implicates non-genomic pathways, particularly GPER-1 and ERα36, in sustaining proliferative and adaptive responses across tumor context [173]. Their co-activation emerges as a plausible mechanistic axis linking signaling plasticity with clinically relevant subtypes and therapeutic resistance [8, 106].

To better integrate these findings into a clinically relevant framework, GPER-1 and ERα36 coexpression can be conceptualized across three functional scenarios:

i. **Luminal endocrine-resistant tumors**, where coactivation of GPER-1/ERα36 sustains MAPK/ERK and PI3K/AKT signaling despite ERα inhibition;
ii. **ERα-negative or triple-negative tumors** with retained estrogen responsiveness mediated by non-genomic receptors;
iii. **Environmentally modulated sporadic tumors**, where ligands such as BPA enhance signaling intensity and pathway activation.

This integrative framework supports the development of combinatorial biomarkers based on non-genomic estrogen receptors (Figure 2). Within this context, co-activation of GPER-1 and ERα36 may amplify proliferative signaling, enhance phenotypic plasticity, and facilitate therapeutic evasion, particularly in aggressive or treatment-resistant tumor subtypes [174, 175].

From a translational perspective, multi-omic profiling strategies integrating genomic and transcriptomic data have demonstrated the ability to refine molecular classification beyond conventional hormone receptor status, enabling tumor stratification with greater functional resolution [176, 177] (Table 1). Notably, transcriptomic analyses from public datasets such as TCGA suggest that GPER expression may be reduced in certain breast cancer subtypes compared to normal tissue [178, 179]. However, this apparent decrease in expression does not necessarily reflect diminished functional activity. Instead, it may indicate context-dependent regulation, including altered receptor localization, differential expression across tumor subpopulations, or compensatory activation of parallel signaling pathways [64, 102, 123, 180]. These observations emphasize the need to integrate expression data with functional analyses, highlighting tumor heterogeneity and supporting dynamic classification frameworks based on receptor activity and integrated signaling networks.

This approach supports the identification of biomarkers associated with endocrine resistance and may guide patient stratification for targeted or combination therapies involving non-genomic estrogen signaling pathways [67, 162].

Expanding the understanding of estrogenic signaling by incorporating the functional roles of GPER-1 and ERα36 provides a conceptual framework for investigating non-genomic estrogen networks in breast cancer [121, 127]. Within this context, GPER-1 and ERα36 could be incorporated into multi-layer biomarker panels, particularly in subtypes characterized by high plasticity such as luminal B and TNBC [20, 31, 181]. Their inclusion may improve the identification of endocrine-resistant phenotypes and support the development of combination therapies targeting both genomic and non-genomic pathways.

This approach is consistent with emerging precision oncology frameworks, in which integrated signaling networks, rather than individual biomarkers, guide therapeutic decision-making [163, 182]. However, validation in clinically annotated cohorts remains essential to determine their translational relevance and applicability [6].

## Conclusion

In recent years, estrogen signaling in breast cancer has expanded beyond the classical ERα genomic pathway to include non-genomic mechanisms that contribute to tumor heterogeneity. This supports a shift from receptor-centered models toward integrated signaling network frameworks that better reflect tumor biology. Within this context, incorporating GPER-1 and ERα36 into tumor characterization may refine ER-based classification by identifying tumors driven by membrane-initiated estrogen signaling, often underrecognized in clinical practice.

In luminal tumors, particularly luminal B, ERα36 overexpression is associated with endocrine resistance and activation of EGFR, MAPK, and PI3K/AKT pathways. GPER-1 and ERα36 also respond to endocrine disruptors such as BPA, linking environmental exposure to tumor development.

Although supported by experimental evidence, these findings still require validation in prospective clinical cohorts.

This link between environmental exposure and non-genomic estrogen signaling highlights the need to consider environmental factors in breast cancer models.

From a translational perspective, integrating receptor signaling with exposure biology may help explain tumor variability and uncover clinically unrecognized subtypes.

Overall, GPER-1 and ERα36 contribute to signaling plasticity and are associated with aggressive behavior and therapeutic resistance. Their use as functional biomarkers may improve stratification and help predict endocrine resistance. Future studies should validate their combined analysis using multi-omics and spatial approaches, moving toward functional rather than purely descriptive tumor classification.

Taken together, the integration of GPER-1 and ERα36 within a unified framework of non-genomic estrogen signaling provides a conceptual basis for understanding how environmental cues, receptor plasticity, and intracellular signaling networks converge to shape breast cancer progression and therapeutic response.

## Abbreviations

BPA: bisphenol A
E1: estrone
E2: estradiol
E3: estriol
EDCs: endocrine-disrupting chemicals
EMT: epithelial-mesenchymal transition
ERα: estrogen receptor alpha
ERβ: estrogen receptor beta
EVs: extracellular vesicles
GPCR: G protein-coupled receptor
GPER-1: G protein-coupled estrogen receptor 1
SERMs: selective estrogen receptor modulators
TMX: tamoxifen
TNBC: triple-negative breast cancer

## Declarations

### Copyright

© The Author(s) 2026.
